# Large genomic rearrangements in BRCA1 and BRCA2 genes in breast and ovarian cancer families in Poland

**DOI:** 10.1186/1897-4287-10-S1-A1

**Published:** 2012-01-12

**Authors:** Helena Bielecka, Bohdan Górski

**Affiliations:** 1 International Hereditary Cancer Center, Department of Genetics and Pathology, Pomeranian Medical University, Szczecin, Poland

## 

Mutations in the BRCA1 and BRCA2 genes predispose women to breast and ovarian cancer. The large majority of the alterations identified in these genes are point mutations and small insertion/deletion. However, an increasing number of large genomic rearrangements are being identified, especially in BRCA1. To date 161 and 39 gene alterations have been described in the literature, approximately for BRCA1 and BRCA2. Just few large genomic rearrangements of BRCA1 gene have been reported in Poland.

Technical limitations of conventional PCR-based methods are cause that gross rearrangements can be overlooked. It has been suggest that about 30% of mutations in the BRCA1 gene are missed by standard mutation detection methods. We use Multiplex Ligation-dependent Probe Amplification (MLPA) to analyze BRCA1/2 rearrangements in 300 unrelated patients with strong family history of breast and/or ovarian cancer negative for BRCA1 Polish founder mutation.

The purpose of this study is establish the prevalence of BRCA1 and BRCA2 large genomic rearrangements in patients with hereditary breast and/or ovarian cancer of Polish population.

